# Changes in food choice attitudes and body image perception at two yrs. post-bariatric surgery

**DOI:** 10.1186/1758-5996-7-S1-A140

**Published:** 2015-11-11

**Authors:** Liane Murari Rocha, Mariana Barbosa Boiani, Daniela Cristina Silva Baldan, Elinton Adami Chaim, Sarah Monte Alegre

**Affiliations:** 1 Universidade Estadual de Campinas, Campinas, Brazil

## Background

Food choices are dynamic and partly determined by cultural, environmental and psychosocial factors. These factors can also influence the construction of a body image but are likely affected by bariatric surgery.

## Objective

To assess possible changes in food choices and body image of women two yrs. after bariatric surgery.

## Materials and methods

We evaluated 37 women outpatients of the Hospital of Unicamp, divided into two groups: preoperative (PreOp, n=16) and postoperative of gastric bypass Y Roux (PosOp, n=21). The mean age was 36.7±7.8 and 40.7±9.3 yrs. and BMI 47.2±3.8 and 26.7±3.3 kg/m2, respectively. Food choices were evaluated by Taste Attitude Scale, divide into sub-scales: craving for sweet foods, using food as a reward and pleasure. Body image was assessed by Silhouettes Scale for Brazilian Adults, both validated instruments. The anthropometric measurements included current weight and height. Data were analyzed using SPSS v.20, used the Mann-Whitney and Wilcoxon tests, p < 0.05.

## Result

Weight loss was 43.1±8.3% of the initial weight in the PosOp group. About food choices, there was no difference to craving for sweet foods (PreOp 8.5±3.9 vs PosOp 8.9±3.1) and pleasure (PreOp 19.7±2.5 vs PosOp 18.7±4,4), only for the using of food as a reward (Preop 18.4±4.9 vs 14.5±4.4 PosOp; p=0.025).The perception of body image did not differ (Preop -0.13±1.8 vs 0.1±2.3 PosOp), but the dissatisfaction differed (PreOp 7.6±1.6 vs PosOp 1.9±2.3 ; p=0.001). There was a difference in the picture considered healthy (PreOp 26.8±4.7 vs PosOp 21.6±4.6 kg/m2; p=0.006) and the desired image (PreOp 26.6±3.2 vs PósOp 20.9±5.5 kg/m2; p=0.001), with no difference between these intragroup.

## Conclusion

At two yrs. post-surgery, the patients had not relinquished their sweet cravings and food-for-pleasure attitudes, but the use of food as a reward was diminished. They had good body awareness and were less dissatisfied with their own body looks, although they considered the healthy and idealized image to be still thinner. Psychological support could be important to prevent the development of eating disorders.

**Figure 1:**
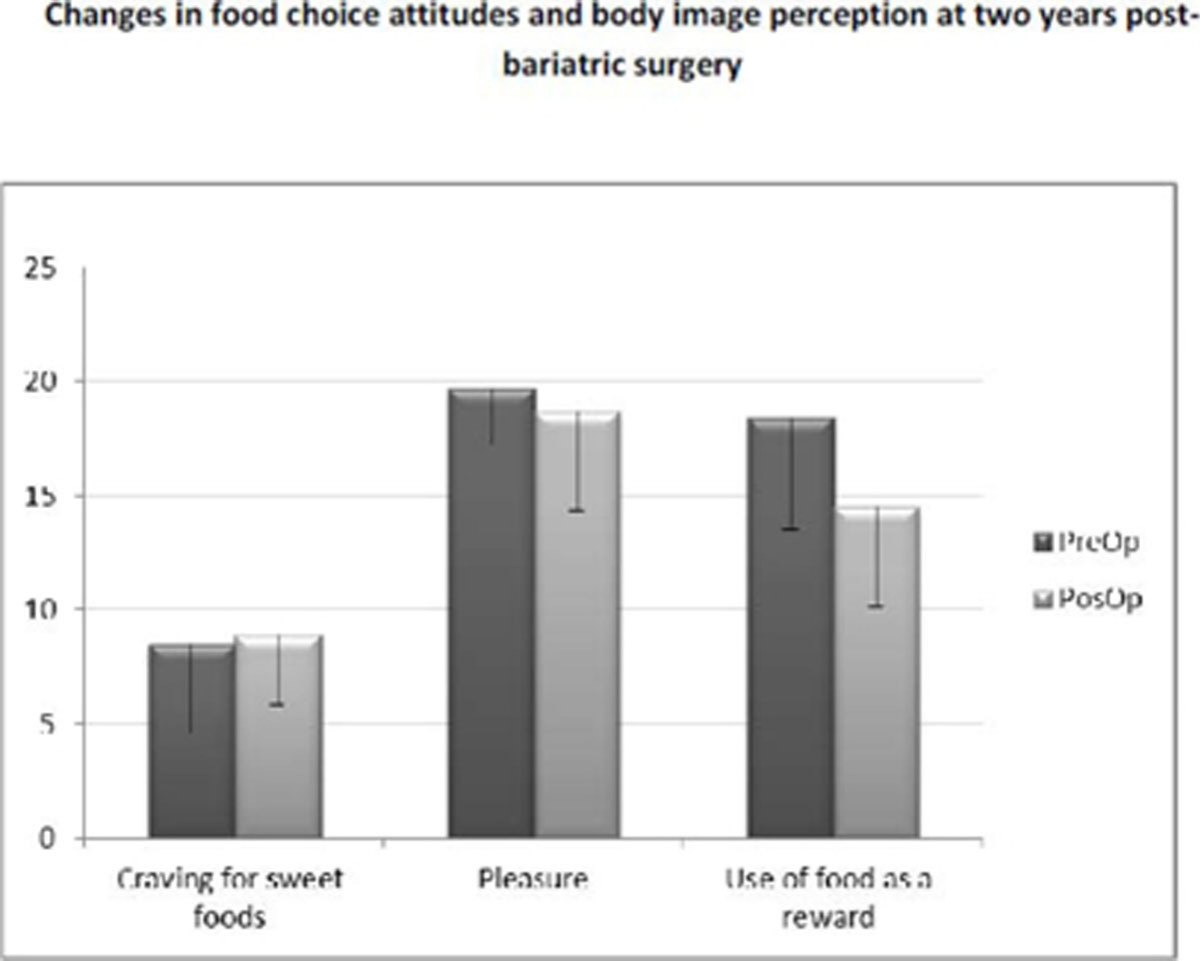
Food choices attitudes before and after bariatric surgery.

**Figure 2:**
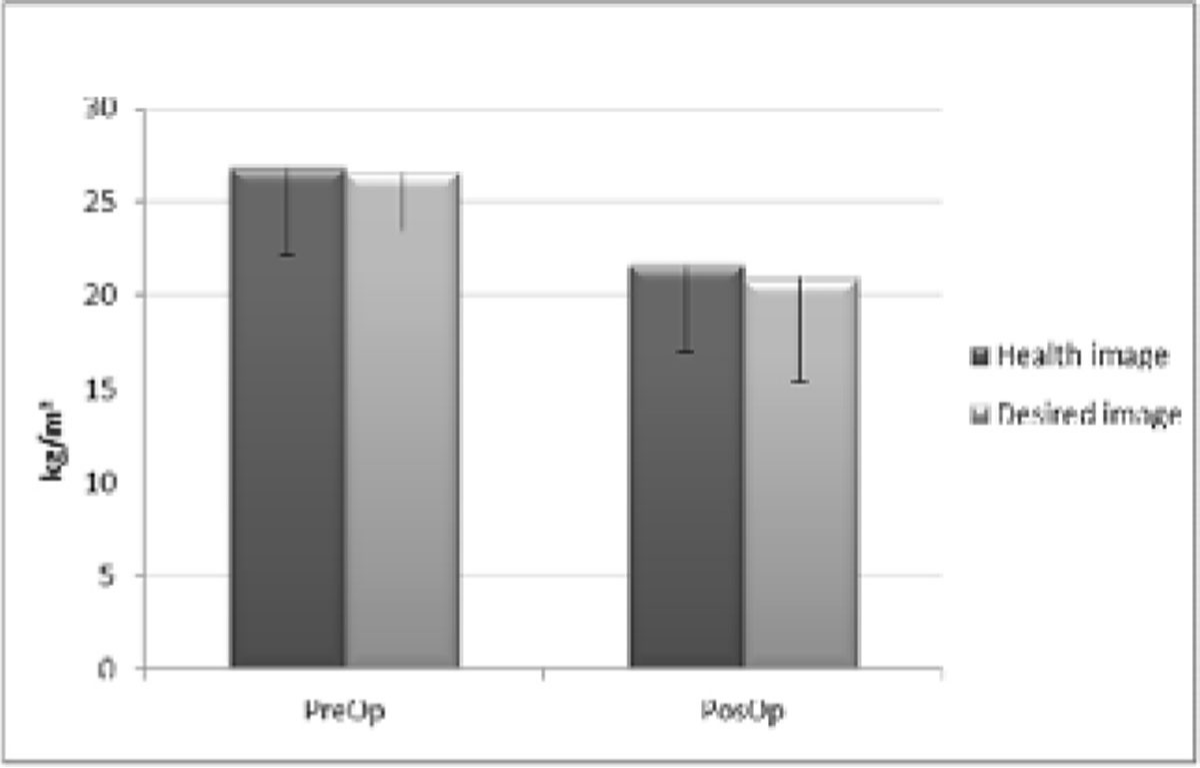
Body image before and after bariatric surgery.

